# Electroacupuncture Ameliorates the Coronary Occlusion Related Tachycardia and Hypotension in Acute Rat Myocardial Ischemia Model: Potential Role of Hippocampus

**DOI:** 10.1155/2015/925987

**Published:** 2015-06-29

**Authors:** Shengbing Wu, Jian Cao, Tianning Zhang, Yiping Zhou, Keming Wang, Guoqi Zhu, Meiqi Zhou

**Affiliations:** ^1^Key Laboratory of Xin'an Medicine, Ministry of Education, Anhui University of Chinese Medicine, Hefei 230038, China; ^2^Department of Science and Technology, Anhui University of Chinese Medicine, Hefei, China; ^3^Anhui No. 2 Province People's Hospital, Hefei, China; ^4^Institute of Acupuncture and Meridian, Anhui University of Chinese Medicine, Qianjiang Road 1, Hefei 230012, China

## Abstract

Mechanisms for electroacupuncture (EA) in disease treatments are still enigmatic. Here, we studied whether hippocampus was involved in the protection of EA stimulation on myocardial ischemia injury. Acute myocardial ischemia (AMI) model was produced. EA stimulation at heart meridian from Shenmen (HT7) to Tongli (HT5) was applied to rats 3 times a day for continuous three days. Coronary occlusion related tachycardia and hypotension, indicated by heart rate, mean arterial pressure, and rate pressure product, were apparently impaired after AMI injury. By contrast, EA stimulating could ameliorate the impairments of heart function (*P* < 0.05). Interestingly, lesion of CA1 region of hippocampus abolished the protection of EA. Neuronal activity in CA1 area was affected by AMI. As evidenced, cell counts, cell types, and frequency of the discharged neurons were facilitated after AMI, while EA stimulation attenuated the abnormalities. Furthermore, c-Fos expression was significantly facilitated in CA1 area after AMI, which was reduced by EA stimulation. Correlations were established between c-Fos expression and cell counts of discharged neurons, as well as between heart function and cell counts of discharged neurons. Taken together, EA stimulation at heart meridian protects against heart dysfunction induced by AMI possibly through suppressing the neuronal activity in CA1 region.

## 1. Introduction

Acute myocardial ischemia (AMI) is a severe injury to the cardiac muscle cells due to rapid disruption of coronary artery [1]. Myocardial cell necrosis is apparent after ischemia injury, leading to impairment of myocardial function. Clinical manifestations consist of sustained retrosternal pain and chemical changes such as increase of white blood cells and serum myocardial necrosis markers. The morbidity and mortality elicited by myocardial vascular diseases, especially by heart diseases, increase annually worldwide and onset of heart disease has a trend to be much younger. With the coming of aging society in China, cardiovascular disease, therefore, has become the most important public health concern.

Acupuncture, one of the key components of traditional Chinese medicine (TCM), has a long history of experience in clinical practice in China. However, how acupuncture functions in disease treatments is still largely unknown. Acupuncture especially has specific functions in the treatment of ischemic heart diseases [2–5]. A variety of mechanisms likely explain the therapeutic effect of acupuncture on ischemic heart disease, including decreasing lipid peroxidation, facilitating energy metabolism and myocardial enzyme activity, influencing cellular ultrastructure, inhibiting apoptosis, improving cell genesis, and reducing myocardial oxygen demand [6–10]. Scientists also implicated the potential roles of central nervous system and peripheral nervous system involved in the protection of acupuncture in myocardial ischemia injury [11, 12].

Hippocampus belongs to the edge of the forebrain cortex structure. It was interesting to find that electrical stimulation at hippocampus could alter blood pressure, pulse, and breath rate [13–17]. Cardiovascular response caused by hippocampus stimulation was through both vagus nerve and sympathetic nerve [12, 18]. On the basis of those evidences, hippocampus is probably involved in the regulation of myocardial functions by electroacupuncture (EA) stimulation. In this study, experimental approaches such as electrophysiological technique and immunohistochemical assaying were utilized to verify the function of hippocampus involved in the protection of acupuncture and its mechanisms. Through this study, a new avenue underlying the protection of acupuncture was demonstrated.

## 2. Materials and Methods

### 2.1. Reagents

Kainic acid (KA, Sigma); chloral hydrate (Guo Yao Group Chemical Reagent Co., Ltd., China); primary antibody for c-Fos (Beijing Bioss Technology Co., Ltd., China); and the second antibody of universal type by two-step method (Beijing ZSGB-BIO Technology Co., Ltd., China) were used.

### 2.2. AMI Model and Treatments

SD rats (clean grade; weight: 200–250 g) were provided by the Animal Center of Anhui Medical University (License number: SCXK (Wan) 2011-002). All procedures in this study were approved by the committee of Anhui University of Chinese Medicine. The temperature in cages was set to 22 ± 1°C with a relative humidity of 60%. Rats were adapted for at least one week for the subsequent experiments and randomly divided into five groups: sham-operated group, myocardial ischemia injury, myocardial ischemia injury + acupuncture group, hippocampus lesion + myocardial ischemia injury + acupuncture group, and sham-operated + acupuncture group. The AMI model was produced by referring to the literature [19]. Briefly, coronary artery ligation method was applied to the left anterior and the success of AMI model was evaluated by electrocardiogram and 2,3,5-triphenyltetrazolium chloride (TTC) staining (see Supplementary Figure 1 of the Supplementary Material available online at http://dx.doi.org/10.1155/2015/925987).

### 2.3. Lesion of Hippocampus

**Figure 1:**
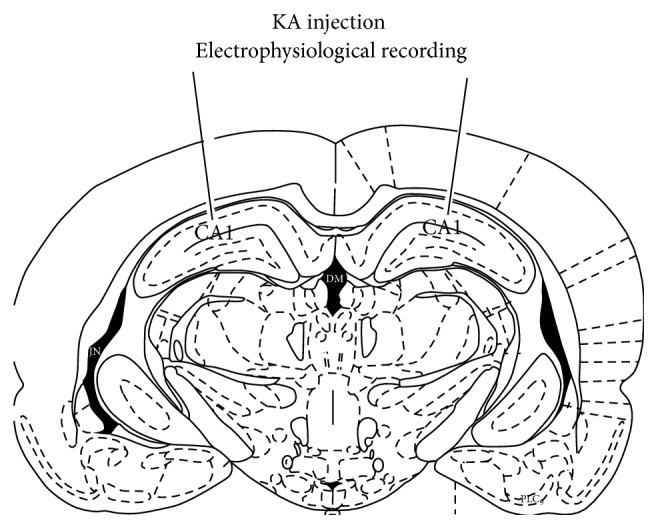
A schematic indicates the site for KA injection and electrophysiological recording in CA1 area.

Intrahippocampal injection of KA (dose: 1 mg/mL, Bregma −3.4 mm, LR 2.4 mm, H 2–2.7 mm) was applied to damage region I of hippocampus proper (CA1) of hippocampus (Figure 1). Three days after surgery, apparent cell death was observed in hippocampal CA1 area (Supplementary Figure 2).

### 2.4. Meridian Selection and Electroacupuncture Parameter

Acupoints at heart meridian from Shenmen (HT7) to Tongli (HT5) were chosen. After anesthesia with 10% chloral hydrate (3.5 mL/kg), rats were positioned on a DC heating pad with a temperature of 36-37°C. EA stimulation was conducted in lesion + acupuncture group, model + acupuncture group, and sham-operated + acupuncture group, respectively, using three 1-inch needles in each meridian section, spacing about 2 mm. The needles were connected to SDZ-IV Electronic Acupuncture Treatment Instrument (Chinese Medicine Apparatus, Jiangxi, China). The EA parameter was set at two bursts of stimulation with a current of 1.1 mA at 2 Hz (duration: 30 min). The EA therapy was initiated at day 1 after surgery for consecutive three days. Rats in model group and sham-operated group deserved sham stimulations (with instrument switch-off).

### 2.5. Cardiovascular Assessments

Two catheters filled with saline were implanted into the femoral artery and femoral vein of rats that were intraperitoneally (*i.p.*) injected with chloral hydrate (3.5 mL/kg). The temperature of the rats was kept at 36-37°C. Catheters were connected to the PowerLab Multipurpose Polygraph Records' Amplifier (AD Instruments, Australia). Heart rate (HR), mean arterial pressure (MAP), and rate pressure product (RPP) were recorded three days after the last acupuncture stimulation.

### 2.6. Electrophysiological Records

Eight-channel nickel alloy electrode through brain stereotaxic instrument was fixed at the brain with parameters of Bregma −3.4 mm, LR 2.4 mm, and H 2–2.7 mm. In our preliminary experiment, a glass electrode with crystal violet was positioned based on the parameter to make sure of the accuracy of exact location (Figure 1). Eight-channel physiological signal in stereotrode or tetrode format was recorded and amplified by OmniPlex amplifier as previously demonstrated [20]. The stereotrode or tetrode bundles were advanced slowly toward the hippocampal CA1 region until the tips of electrodes had reached the CA1 as deduced from an assessment of field potential and neuronal activity patterns. The following parameters were set to record (frequency: 1000 Hz; gain: 5000; and signal-to-noise ratio: 4 : 1) spike wave, action potentials, and local field potentials. The recorded spike activities from those neurons were processed in the manner as previously described [21]. Artifact waveforms were removed and the spike waveform minima were aligned using the Offline Sorter 2.0 software. Cluster analysis was completed based on the input of the Plexon system. Expectation-maximization based competitive mixture decomposition algorithm was applied to distinguish discharged neuronal types as previously demonstrated [22].

### 2.7. Immunohistochemistry

Three days after last EA stimulation, c-Fos expression in CA1 area of hippocampus was measured. The tissues were fixed in 4% paraformaldehyde overnight, cryoprotected in 30% sucrose for 1 h at 4°C, and sectioned on a freezing microtome at 20 *μ*m. After that, the sections were incubated with c-Fos antibody (1 : 100) overnight at 4°C. The immunohistochemistry assay kit (Zhongshan Jianqiao Tech, Beijing, China) was applied to complete the staining. The images were captured using Olympus microscope (Japan). c-Fos positive cells in CA1 area were counted.

### 2.8. Statistical Methods

The data were presented as mean ± standard deviation (SD). Statistical analysis was conducted by SPSS 17.0 software. One-way ANOVA followed by Newman-Keuls post hoc test was applied to determine statistical significance. *P* < 0.05 indicated the significant difference.

## 3. Results

### 3.1. Hippocampus Is Involved in the Protective Effect of Acupuncture

**Figure 2:**
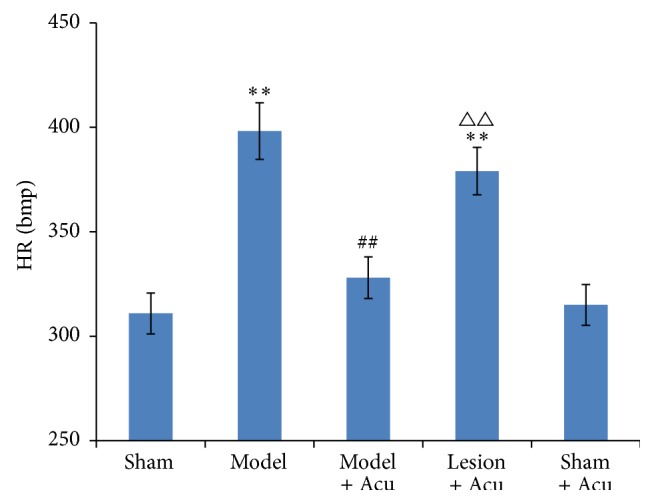
Myocardial ischemia injury impairs heart rate, while being ameliorated by EA treatment. Hippocampus lesion abolished the protection. Data were presented as mean and SD. ^*∗∗*^ *P* < 0.01 compared with sham control. ^##^ *P* < 0.01 compared with model. ^△△^ *P* < 0.01 compared with model + acupuncture group.

Three days after last acupuncture, HR, MAP, and RPP were measured in different groups. Compared with sham-operated group, heart rate was obviously speeded up in AMI model group (*P* < 0.01) (Figure 2). By contrast, EA therapy significantly attenuated AMI-induced elevation of heart rate. Interestingly, lesion of hippocampus partially but significantly mitigated the protection of EA. As a control, acupuncture alone did not affect the heart rate in sham-operated group.

**Figure 3:**
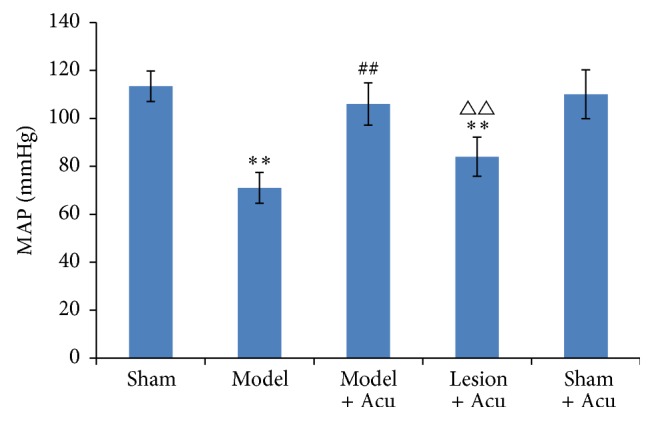
Myocardial ischemia injury impairs mean arterial pressure, while being ameliorated by EA treatment. Hippocampus lesion abolished the protection. Data were presented as mean and SD. ^##^ *P* < 0.01 compared with model. ^△△^ *P* < 0.01 compared with model + acupuncture group.

**Figure 4:**
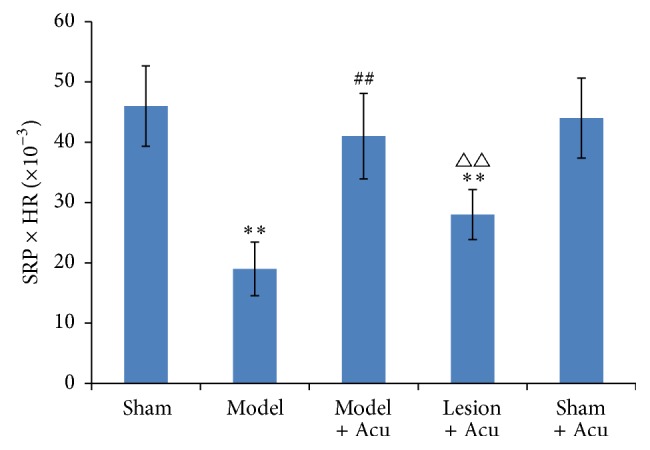
Myocardial ischemia injury impairs rate pressure product, while being ameliorated by EA treatment. Hippocampus lesion abolished the protection. Data were presented as mean and SD. ^##^ *P* < 0.01 compared with model. ^△△^ *P* < 0.01 compared with model + acupuncture group.

Compared with the sham-operated group, MAP was reduced significantly in AMI model group (*P* < 0.01) (Figure 3). Acupuncture therapy at heart meridian attenuated the decrease of MAP. Similar to the heart rate, protective effect of acupuncture on MAP was also partially mitigated by hippocampus lesion. A similar trend was found with RPP (Figure 4); the protective effect of acupuncture on ischemia model was mitigated by hippocampus lesion. Based on the results of HR, MAP, and RPP, we conclude that hippocampus is involved in the protective effects of acupuncture against heart dysfunctions in AMI model.

### 3.2. Acupuncture Attenuates AMI-Induced CA1 Neuronal Activation

**Figure 5:**
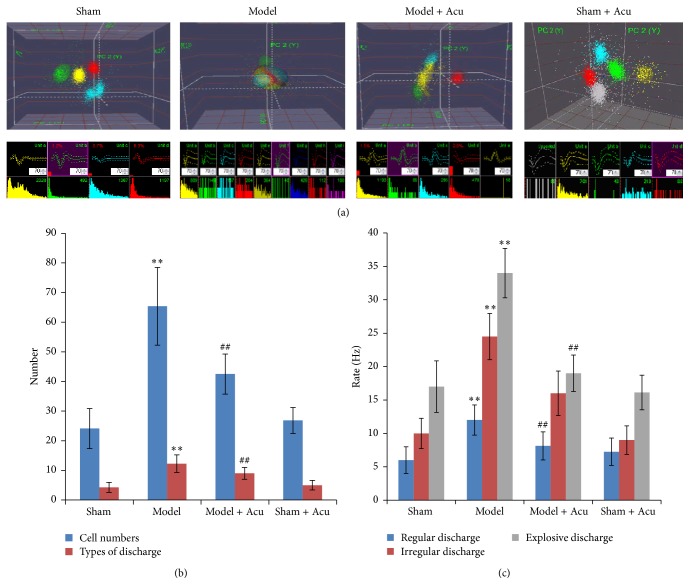
Myocardial ischemia injury activates neuronal discharge, while being ameliorated by EA treatment. (a) Representative electrophysiological images from different groups. (b) Cell numbers and cell types for the discharged cells. (c) Discharge frequency in different groups. Data were presented as mean and SD. ^*∗∗*^ *P* < 0.01 compared with sham control. ^##^ *P* < 0.01 compared with model.

As hippocampus was involved in the protective effect of acupuncture on myocardial ischemia injury, we identified the potential effects of AMI injury or EA on hippocampal CA1 neuronal activity. Both cell types and frequency of discharged neurons were evaluated. Compared with sham-operated group, the types and numbers of the discharged cells in CA1 region were significantly increased in the AMI modeled rats (Figures 5(a) and 5(b)). EA stimulation significantly weakened the neuronal discharge in both cell types and cell numbers.

The neuronal discharge frequency in hippocampal CA1 region was also evaluated. When compared with sham-operated group, the frequencies of regular, irregular, and explosive discharges in CA1 area of hippocampus in model group were significantly increased (*P* < 0.01) (Figures 5(a) and 5(c)). However, when compared with AMI group, the discharged frequencies were significantly lowered after acupuncture stimulation (*P* < 0.01).

### 3.3. Acupuncture Attenuated AMI-Induced c-Fos Expression in Hippocampal CA1 Region

**Figure 6:**
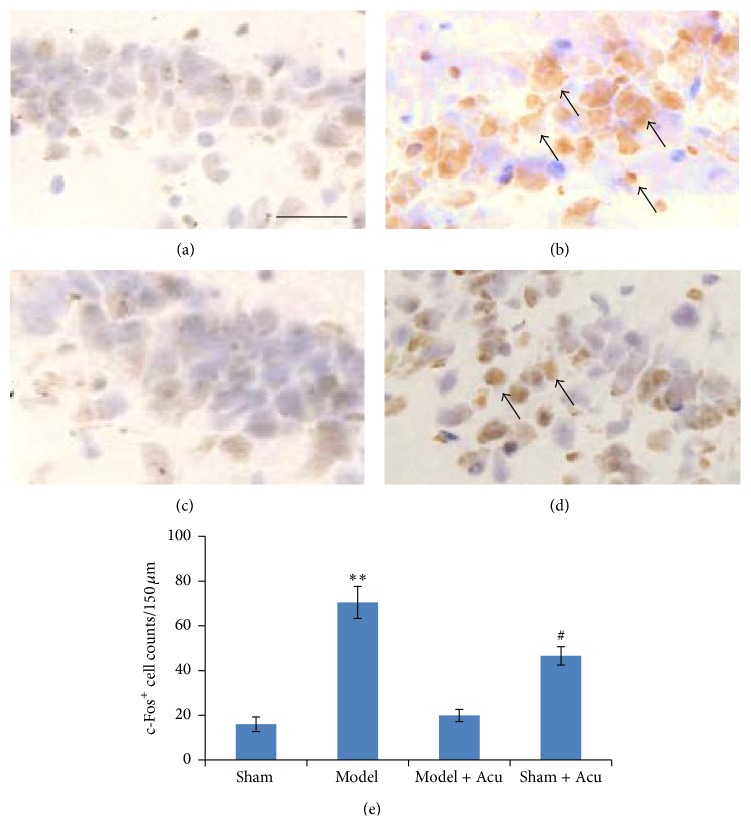
c-Fos expression in CA1 region was activated after myocardial ischemia injury, while being decreased by EA treatment. Representative images for sham (a), model (b), model + acupuncture (c), and sham + acupuncture (d). (e) Quantification data for the c-Fos positive cell numbers. Data were presented as mean and SD. ^*∗∗*^ *P* < 0.01 compared with sham control. ^#^ *P* < 0.05 compared with model.

When compared with sham-operated group, c-Fos positive cells in CA1 region of hippocampus were significantly increased in AMI model group (*P* < 0.01) (Figures 6(a), 6(b), 6(c), 6(d), and 6(e)). By contrast, when compared with model group, c-Fos positive cells in CA1 area were attenuated after EA stimulation (*P* < 0.01). In the sham-operated rats, EA did not affect c-Fos expression.

### 3.4. Correlation Analysis

**Figure 7:**
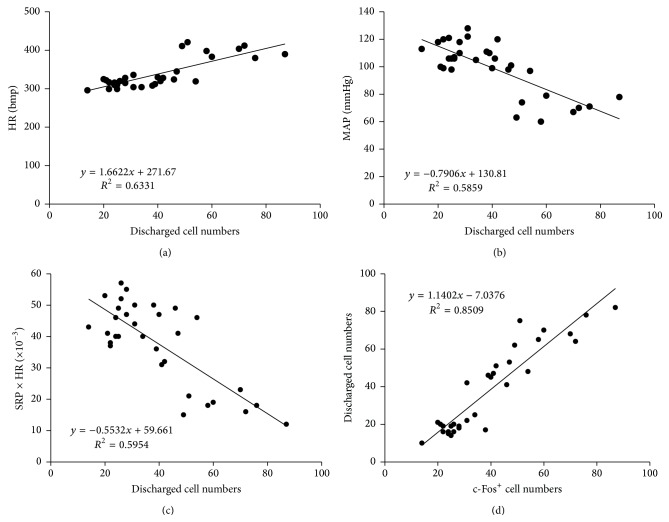
Correlation analysis. (a) Positive correlation between c-Fos positive cells and discharged cell numbers in CA1 region; (b) positive correlation between HR and discharged cell numbers; (c) negative correlation between MAP and discharged cell numbers; and (d) negative correlation between RPP and discharged cell numbers.

The correlations between heart function and hippocampal neuronal discharges were analyzed. As shown in Figure 7(a), a positive correlation between HR and discharged cell numbers in hippocampus was established (*R* ^2^ = 0.6331) while negative correlations were reflected between MAP or RPP and discharged cell numbers (MAP: *R* ^2^ = 0.5859, RPP: *R* ^2^ = 0.5954) (Figures 7(b) and 7(c)). We also analyzed the correlation between the numbers of discharged cells and c-Fos expression. As shown in Figure 7(d), a positive correlation between c-Fos positive cell numbers and discharged cell counts was established (*R* ^2^ = 0.8509).

## 4. Discussion

Acupuncture may perform a role in the treatment of AMI injury [2–4]. In this study, we firstly disclosed a mechanism underlying the protection of acupuncture, that is, acupuncture functions through hippocampus to protect the myocardial function. The correlation between meridian and brain function bridges the Chinese medicine with Western medicine [23]. It is popular nowadays to investigate the functions of channel-viscera in cerebral-related diseases. Based on our studies and in combination with the advance of nerve anatomy, the present study proposed that the effects of acupuncture on myocardial ischemia injury were through regulation of hippocampus-mediated autonomic nervous system.

Initially, we confirmed the functional activity of EA stimulating at heart meridian in ameliorating myocardial injury. HR, MAP, and RAP were impaired in AMI model, which were ameliorated by EA stimulating at heart meridian from Shenmen to Tongli. Previously, EA at bilateral Epangxian I [7] and Neiguan [6] was also reported to prevent myocardial ischemia injury. In those studies, sympathetic discharges were activated after EA [7]. Interestingly, in our present study, we found that lesion of CA1 region by KA abolished the protection of acupuncture. These data firstly point the importance of hippocampus, especially CA1 region in the protection of EA in myocardial ischemia injury.

As reported, myocardial ischemia injury was related to both sympathetic nerve system and vagus nerve system [12]. Under normal physiological condition, a dynamic balance between these two systems sustains the heart function. However, under pathological condition, ischemia injury might signal to suppress vagus system, which will facilitate the sympathetic activity. The phenotypes of this imbalance were displayed by the abnormality of coronary occlusion related tachycardia and hypotension.

In support of our findings, we used a microarray electrophysiological technique to detect the neuronal activity in CA1 region. Myocardial ischemia injury significantly increased the types and numbers of the discharged cells in CA1 region as well as the discharged frequency. Indeed, myocardial function tightly correlates with hippocampal activity. For example, myocardial infarction induces cognitive impairment by increasing the production of hydrogen peroxide in adult rat hippocampus [24]. Acupuncture therapy significantly weakened the neuronal discharge in both cell types and cell numbers, as well as discharged frequencies in modeled rats. Those results implicated that myocardial injury-induced activation of hippocampus neurons was suppressed by EA stimulation. Although the afferent inputs from the acupuncture points to the brain are still not totally clear, we found a functional response of hippocampal neurons to EA stimulation. Our study provides the electrophysiological evidence indicating a central node effect of hippocampus after myocardial ischemia injury or EA. Recently, the real-time effects of EA on hippocampal neuronal activation are being studied in our group. In addition, EA stimulation at subspecific acupoint affected brain glucose metabolism [25].

Besides the electrophysiological alterations, ischemia injury also induced widely expression of c-Fos in CA1 cell body layer. A variety of stimuli, including serum, growth factors, tumor promoters, cytokines, and UV radiation, induce the expression of c-Fos [26–29]. As reported, c-Fos was also involved in important cellular events, including regulating cell proliferation, differentiation, and survival and genes associated with hypoxia and angiogenesis [30]. Importantly, dysfunction of c-Fos protein was also reported to regulate cell polarity [31]. In our study, we found a huge increase of c-Fos expression after myocardial injury, while acupuncture mitigated the expression of c-Fos. These results might implicate that EA suppressed the c-Fos protein expression to reduce activation of hippocampal neurons. Although direct evidence showing the regulation of c-Fos to the neuronal discharge is not demonstrated, a correlation was established after analyzing the c-Fos expression with the discharged cell numbers in hippocampus. This result was consistent with the previous literature [32].

**Figure 8:**
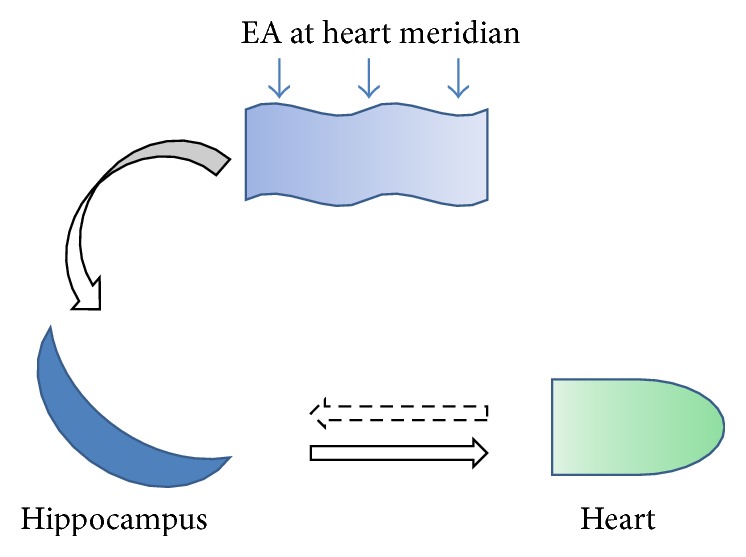
A schematic illustrates the potential function of hippocampus involved in the protection of EA stimulation at heart meridian on AMI-induced heart dysfunction. AMI injury was reflected by the activation of hippocampal CA1 neurons. Reversely, EA stimulation could send signal to hippocampus and inhibit the activation of neurons. Subsequently, the balance between sympathetic nerve system and vagus nerve system was reestablished to ameliorate heart function.

Another important question is how hippocampus is regulated by EA to ameliorate heart function. Heart function was regulated by both sympathetic nerve system and vagus nerve system. In our initial hypothesis, we suggested that different cerebral nuclei send signals to sympathetic nerve system and vagus nerve system. A new balance will be established between sympathetic nerve system and vagus nerve system after EA, finally ameliorating heart function (Figure 8). This point will be continually studied in the future.

## 5. Conclusion

In summary, this study provides a new pathway underlying the ameliorating effects of EA on myocardial ischemia injury. Hippocampus, especially the CA1 area functions as an important regulator in the effects of EA. In addition, activation of hippocampal neurons was suppressed by EA, likely through regulating c-Fos expression. We demonstrated an important role of central nerve system in the functions of EA in the disease treatment.

## Supplementary Material

Coronary artery ligation method was applied to the left anterior and the success of Acute myocardial ischemia (AMI) model was evaluated by electrocardiogram and HE staining. As shown in sFig.1, typical injury found in AMI groups. The lesion of CA1 was carried out by injection of KA in CA1 area. After injection, the rats were decapitated and HE staining was used to detect cell death (sFig.2).

## References

[1] Ohman E. M., Armstrong P. W., Christenson R. H. (1996). Cardiac troponin T levels for risk stratification in acute myocardial ischemia. GUSTO IIA Investigators. *The New England Journal of Medicine*.

[2] Shen H., Chen Y. J. (2012). Effects of electro-acupuncture combined general anesthesia on myocardial injury of high blood sugar patients with coronary heart disease in the perioperative phase. *Chinese Journal of Integrated Traditional and Western Medicine*.

[3] Alraek T., Tan C. O. (2011). Acupuncture and heart rate variability. *Acupuncture in Medicine*.

[4] Yang L., Yang J., Wang Q. (2010). Cardioprotective effects of electroacupuncture pretreatment on patients undergoing heart valve replacement surgery: a randomized controlled trial. *The Annals of Thoracic Surgery*.

[5] Gemma M., Nicelli E., Gioia L., Moizo E., Beretta L., Calvi M. R. (2015). Acupuncture accelerates recovery after general anesthesia: a prospective randomized controlled trial. *Journal of Integrative Medicine*.

[6] Redington K. L., Disenhouse T., Li J. (2013). Electroacupuncture reduces myocardial infarct size and improves post-ischemic recovery by invoking release of humoral, dialyzable, cardioprotective factors. *The Journal of Physiological Sciences*.

[7] Li M. P., Sun G. J., You X. H. (2012). Effects of electroacupuncture stimulation of scalp-point on cardiac sympathetic discharges, myocardial beta1-adrenoceptor protein expression and plasma norepinephrine concentration in myocardial ischemia-reperfusion injury rats. *Acupuncture Research*.

[8] Zhang J., Jia X.-H., Xu Z.-W. (2013). Improved mesenchymal stem cell survival in ischemic heart through electroacupuncture. *Chinese Journal of Integrative Medicine*.

[9] Zhou W., Ko Y., Benharash P. (2012). Cardioprotection of electroacupuncture against myocardial ischemia-reperfusion injury by modulation of cardiac norepinephrine release. *The American Journal of Physiology—Heart and Circulatory Physiology*.

[10] Li P., Pitsillides K. F., Rendig S. V., Pan H.-L., Longhurst J. C. (1998). Reversal of reflex-induced myocardial ischemia by median nerve stimulation: a feline model of electroacupuncture. *Circulation*.

[11] Li Z., Wang C., Mak A. F. T., Chow D. H. K. (2005). Effects of acupuncture on heart rate variability in normal subjects under fatigue and non-fatigue state. *European Journal of Applied Physiology*.

[12] Hu X., Yang X., Jiang H. (2012). Role of sympathetic nervous system in myocardial ischemia injury: beneficial or deleterious?. *International Journal of Cardiology*.

[13] Ruit K. G., Neafsey E. J. (1988). Cardiovascular and respiratory responses to electrical and chemical stimulation of the hippocampus in anesthetized and awake rats. *Brain Research*.

[14] Tjen-A-Looi S. C., Guo Z.-L., Li M., Longhurst J. C. (2013). Medullary GABAergic mechanisms contribute to electroacupuncture modulation of cardiovascular depressor responses during gastric distention in rats. *American Journal of Physiology—Regulatory, Integrative and Comparative Physiology*.

[15] Tjen A. L. S. C., Guo Z. L., Longhurst J. C. (2014). GABA in nucleus tractus solitarius participates in electroacupuncture modulation of cardiopulmonary bradycardia reflex. *The American Journal of Physiology—Regulatory, Integrative and Comparative Physiology*.

[16] Tjen-A-Looi S. C., Hsiao A.-F., Longhurst J. C. (2011). Central and peripheral mechanisms underlying gastric distention inhibitory reflex responses in hypercapnic-acidotic rats. *The American Journal of Physiology—Heart and Circulatory Physiology*.

[17] Tjen-A-Looi S. C., Li P., Li M., Longhurst J. C. (2012). Modulation of cardiopulmonary depressor reflex in nucleus ambiguus by electroacupuncture: roles of opioids and gamma-aminobutyric acid. *The American Journal of Physiology—Regulatory Integrative and Comparative Physiology*.

[18] Coiado O., Buiochi E., O’brien W. (2015). Ultrasound-induced heart rate decrease: role of the vagus nerve. *IEEE Transactions on Ultrasonics, Ferroelectrics, and Frequency Control*.

[19] House S. L., Wang J., Castro A. M., Weinheimer C., Kovacs A., Ornitz D. M. (2015). Fibroblast growth factor 2 is an essential cardioprotective factor in a closed-chest model of cardiac ischemia-reperfusion injury. *Physiological Reports*.

[20] Lin L., Chen G., Xie K., Zaia K. A., Zhang S., Tsien J. Z. (2006). Large-scale neural ensemble recording in the brains of freely behaving mice. *Journal of Neuroscience Methods*.

[21] Lin L., Osan R., Shoham S., Jin W., Zuo W., Tsien J. Z. (2005). Identification of network-level coding units for real-time representation of episodic experiences in the hippocampus. *Proceedings of the National Academy of Sciences of the United States of America*.

[22] Shoham S., Fellows M. R., Normann R. A. (2003). Robust, automatic spike sorting using mixtures of multivariate t-distributions. *Journal of Neuroscience Methods*.

[23] Fu L.-W., Longhurst J. C. (2005). Interactions between histamine and bradykinin in stimulation of ischaemically sensitive cardiac afferents in felines. *The Journal of Physiology*.

[24] Liu C., Liu Y., Yang Z. (2014). Myocardial infarction induces cognitive impairment by increasing the production of hydrogen peroxide in adult rat hippocampus. *Neuroscience Letters*.

[25] Yang M., Yang J., Zeng F. (2014). Electroacupuncture stimulation at sub-specific acupoint and non-acupoint induced distinct brain glucose metabolism change in migraineurs: a PET-CT study. *Journal of Translational Medicine*.

[26] Yamada M., Saitoh A., Ohashi M., Suzuki S., Oka J., Yamada M. (2015). Induction of c-Fos immunoreactivity in the amygdala of mice expressing anxiety-like behavior after local perfusion of veratrine in the prelimbic medial prefrontal cortex. *Journal of Neural Transmission*.

[27] Ay I., Napadow V., Ay H. (2015). Electrical stimulation of the vagus nerve dermatome in the external ear is protective in rat cerebral ischemia. *Brain Stimulation*.

[28] Kwak Y., Han J., Rhyu M., Nam T. S., Leem J. W., Lee B. H. (2015). Different spatial expressions of c-Fos in the nucleus of the solitary tract following taste stimulation with sodium, potassium, and ammonium ions in rats. *Journal of Neuroscience Research*.

[29] Shehab S., D'souza C., Ljubisavljevic M., Redgrave P. (2014). High-frequency electrical stimulation of the subthalamic nucleus excites target structures in a model using *c-fos* immunohistochemistry. *Neuroscience*.

[30] Milde-Langosch K. (2005). The Fos family of transcription factors and their role in tumourigenesis. *European Journal of Cancer*.

[31] Reichmann E., Schwarz H., Deiner E. M. (1992). Activation of an inducible c-FosER fusion protein causes loss of epithelial polarity and triggers epithelial-fibroblastoid cell conversion. *Cell*.

[32] Kovács Z., Puskás L., Nyitrai G. (2007). Suppression of spike-wave discharge activity and c-fos expression by 2-methyl-4-oxo-3H-quinazoline-3-acetyl piperidine (Q5) in vivo. *Neuroscience Letters*.

