# Supplementary material for: Electroacupuncture Ameliorates the Coronary Occlusion Related Tachycardia and Hypotension in Acute Rat Myocardial Ischemia Model: Potential Role of Hippocampus
Source: Evid Based Complement Alternat Med. 2015 Jun 29;2015:925987. doi: 10.1155/2015/925987 (PMC4499623; doi:10.1155/2015/925987)
Supplement: Supplementary file 1 — Coronary artery ligation method was applied to the left anterior and the success of Acute myocardial ischemia (AMI) model was evaluated by electrocardiogram and HE staining. As shown in sFig.1, typical injury found in AMI groups. The lesion of CA1 was carried out by injection of KA in CA1 area. After injection, the rats were decapitated and HE staining was used to detect cell death (sFig.2). [file 925987.f1.docx]

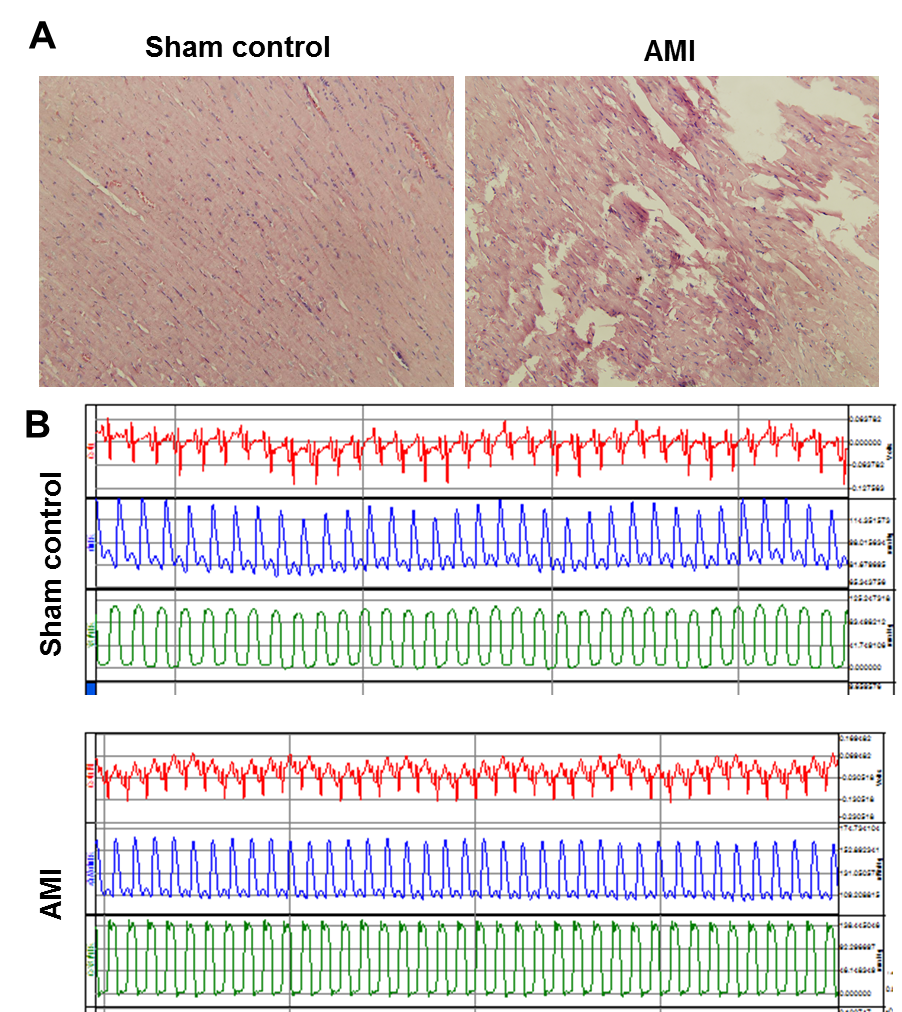


**sFigure.1** AMI model was evaluated by HE staining and ECG. A) Representative images of HE staining. (B) Representative ECG from sham control and ischemia injury group.

**
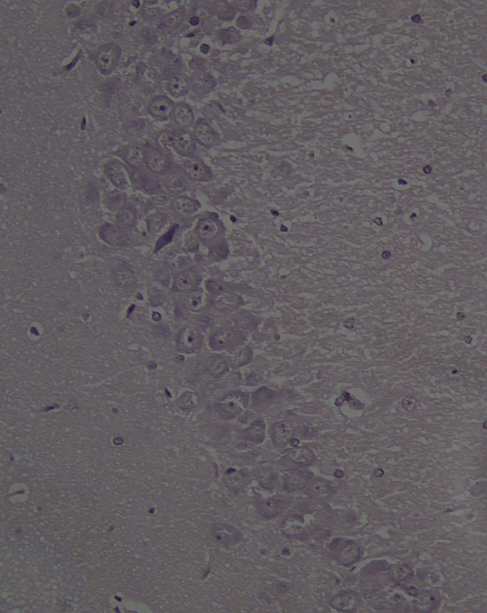
** **
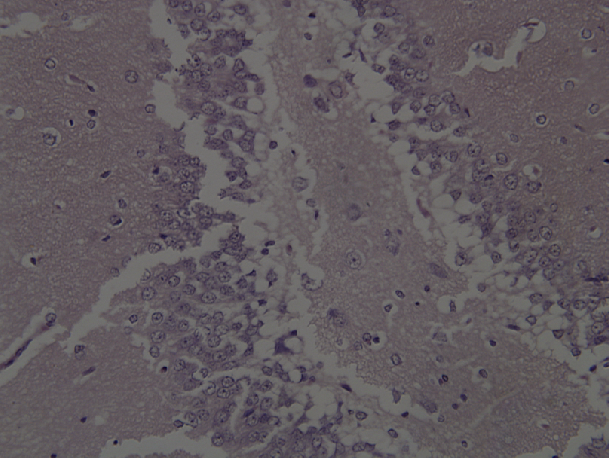
**

1. (B)

**sFigure.2** KA injection damages the CA1 region. (A) Sham control; (B) KA injection.
